# Exploration of facilitators of and barriers to the community-based service utilization for newborn possible serious bacterial infection management in Debre Libanos District, Ethiopia: descriptive qualitative study

**DOI:** 10.1186/s12887-020-02211-9

**Published:** 2020-06-20

**Authors:** Kasahun Girma Tareke, Yohannes Kebede Lemu, Garumma Tolu Feyissa

**Affiliations:** Department of Health, Behavior and Society, Jimma University, P. O. Box 378, Jimma, Ethiopia

**Keywords:** Community-based newborn care, Possible serious bacterial infection, Newborn serious illness, Ethiopia, Barriers

## Abstract

**Background:**

Globally, possible serious bacterial infection [PSBI] is a cause for about 600,000 newborn deaths per year. To decrease the burden of this infection, a community-based management newborn PSBI when referral to hospital is not possible has been on implementation. Studies showed gaps in the service utilization and this study was aimed at exploring its barriers and facilitators.

**Methods:**

A descriptive qualitative study was conducted from March 11– April 7, 2019, in Debre Libanos District, Ethiopia. Study participants were recruited purposively. Women who gave birth within 2 months before data collection, health extension workers [HEW], health workers, religious leader, kebele chairman, and other community members were involved in the study. Five in-depth interviews, seven key informant interviews, and four focused group discussions were conducted with a total of fifty-two participants. The data were audio-recorded, transcribed verbatim and translated, and inductive thematic analysis was done using Atlas ti.7.1 software.

**Result:**

The availability of health workers trained on community-based newborn care [CBNC], Integrated Management of Newborn and Childhood Illness guidelines, availability of medical supplies and job aids, and performance review meetings were identified as facilitators. Communities perception that the newborn illness has no medical treatment, newborn illness is not severe and is self-resolution; the belief in healing power of traditional medicines, socio-cultural and religious beliefs, lack of awareness about service availability at the health post, poor supportive supervision or monitoring, shortage of HEW, the residency of HEWs outside the health post, a poor commitment of health workers and HEWs, and non-functionality of health developmental army were explored as barriers.

**Conclusions:**

The findings provided insight into the facilitators of and barriers to community-based service utilization for newborn PSBI management. There is a need to develop strategies to address the barriers. Therefore, health care providers should have to develop strategies, and conduct a behavioral change communication to change the perception of community members towards newborn illnesses, promote the availability of the service at the health post, and the HEWs provide the service staying at the health post.

## Background

Globally, there were an estimated number of 2.5 million newborn deaths in 2018, mostly from preterm birth, intrapartum complications, and bacterial infection [1]. The bacterial infection, named possible serious bacterial infection [PSBI], is defined as a clinical syndrome used in the Integrated Management of Childhood Illness [IMNCI] package referring to a sick young infant (0–59 months) who requires urgent referral to hospital. The signs are unable to feed or stopped feeding well, convulsions, fast breathing, severe chest in-drawing, fever, low body temperature, movement only when stimulated, or no movement at all [2, 3]. It was caused an estimated number of 6.9 million newborn morbidities [4], and 600,000 newborn deaths per year [3], which is roughly 23% of neonatal deaths, still the proportion is as high as 50% in low-income settings [5, 6]. The incidence ranges from 5.5 to 170 cases/1000 live births for blood culture-confirmed infections and clinically diagnosed cases, respectively [7]. In Ethiopia, it is also a cause for newborn mortality [8]. A prospective study conducted in Ethiopia from 2012 to 2013 showed that 34.3% of neonatal deaths were caused as a result of a neonatal infection [9].

It is most prevalent in low- and middle-income countries particularly in sub-Saharan Africa and Southern Asia [4]. This is because these countries have conditions such as poor quality of care around the time of birth [4], limited attention given for newborns on critical first few days, low institutional delivery and giving birth in settings with suboptimal hygiene and non-sterile techniques [10], premature birth, low coverage of maternal immunization [11], and poor preventive measures [5, 6]. Also, almost 98% of deaths due to this infection occur at these countries [3] due to presence of poor timely care-seeking, limited treatment with appropriate antibiotics or follow up [5, 6], lack of receiving the recommended inpatient treatment due to accessibility, acceptability or affordability problems resulting in unnecessary, potentially preventable infection-related newborn death [3].

To overcome such challenges, the world health organization [WHO] developed a guideline that provides programmatic and clinical guidance and recommends the provision of effective treatment for young infants with severe infection at first-level health facilities to increase access to potentially lifesaving care for these infants when families do not accept or cannot access referral [2, 3]. Besides these, studies indicated that interventions like management of newborn PSBI at the community level by is associated with reduced newborn mortality [6, 12]. Our country, Ethiopia, also adapted the WHO guideline developing the implementation strategies that fit the local context to accelerate the MDG4 achievement and the subsequent agendas to reduce newborn mortality [13, 14]. The country had made remarkable achievements in Millennium Development Goal 4 [MDG4] by reducing under-5 mortality from 205 deaths per 1000 live births in 1990 to 64 deaths per 1000 live births in 2013. However, despite this remarkable progress, newborn mortality was decreased much slower; 55 deaths per 1000 live births in 1990 to 28 deaths 1000 live births in 2013, which accounted for 45% of under-5 mortality [15], and also increased to 30 deaths per 1000 live births in 2019 [16]. Thus, to reduce newborn mortality, Ethiopia piloted the guideline from 2008 and 2013 evaluating the impact of a regimen of intramuscular gentamicin and oral amoxicillin given by HEWs to newborns and young infants with signs of PSBI when a referral is not possible and launched the project on March 2013 [12].

Currently, the service is being delivered as a CBNC package and high impact newborn and child survival intervention focusing on 0–2 month newborns [13, 17]. Under the supervision of primary health care unit [PHCU], trained health extension workers are the frontline service promoters and providers for sick newborns at the community level (both at home and health post [18]. The program also utilizes health developmental armies [HDA] and other existing effective community mobilization mechanisms to scale up the service and to improve maternal and newborn care practices and care-seeking [13]. There is also evidence that showed community-based service utilization is being provided for newborn PSBI management [12, 19, 20]. But, the study findings also showed that there were newborns that did not get treatment service besides the presence of signs or symptoms suggestive to PSBI [12, 19]. Nevertheless, limited information was available on what factors deter or facilitate its service utilization. Addressing the facilitators and barriers for its service utilization at the community level is imperative. Therefore, to address this knowledge gap, the study was utilized descriptive qualitative study to explore potential barriers and facilitators for the community-based service utilization for newborn PSBI management.

## Methods

### Study design, setting and period

This is a descriptive qualitative study that was conducted in Debre Libanos District, North Shoa, Oromia regional state, Ethiopia from March 11– April 7, 2019. It is located 90 km away from Addis Ababa in North direction. There was an estimated number of 64, 305 populations within the District [21] where 77.1 and 22.9% of the population lives in rural and urban, respectively and about 99.29% of them follow the Ethiopian Orthodox Christianity religion [22]. There were also four Health officers, one-degree nurse, twenty clinical nurses, one public nurse, four laboratory technicians, two druggists, five midwifery nurses, fourteen rural health extension workers [HEWs], and five urban HEWs who provide health care service for the populations, and two health centers [HC], ten health posts [HP] and three private primary clinics are there from which the populations of this setting utilize health care service [21].

### Sampling

The study participants were recruited purposively from six kebeles. The kebeles were selected considering the number of catchment kebeles per health center [HC], diversity in distance from health center [HC], rural versus urban residence, and performance of health extension workers [HEWs]. Women who gave birth within 2 months before data collection, women whose newborn died within 2 months of life, parents of a child who got treatment service at HP and HC within the last 2–3 years; care-givers [husbands, mothers and fathers] of women who delivered within 2 months before the data collection, pregnant mothers, mothers-in-law, fathers-in-law, and other reproductive age group peoples, religious leader, and kebele chairman were involved on the study from the community members. Also, from health facilities, health care providers like midwife nurses, clinical nurses who work at under-five clinics, and director of the health center; District Health Office Maternal, Neonatal, and Child health [MNCH] expert, and health extension worker were recruited. Participants from the health facility and community level were recruited based on their role on the implementation of the program activities [i.e. as a monitor or direct implementer], and having rich information on newborn illnesses or their role as a caregiver of newborns, respectively.

### Data collection procedures (instrument, personnel, data collection)

A total of five in-depth interviews, seven key informant interviews, and four focused group discussions [FGDs] were conducted with a total of fifty-two participants. Four women who gave birth within 2 months before data collection and a woman whose newborn was died within 2 months of life participated in an in-depth interview [IDI] face to face. The key informants were religious leader, HEW, kebele chairman, midwife-nurse, U-5 clinic focal, director of the health center, and District health office Maternal, Neonatal, and Child health care [MNCH] expert. A total of 7–12 individuals participated, seated circular, in each FGD with a total of ten women who gave birth within 2 months before data collection and thirty other participants mentioned above.

Data were collected using a semi-structured guide (6–10 open-ended questions customized as per the respondent type) which was developed first in the English language and then translated into Afan Oromo and Amharic languages and back-translated into English by an independent translator. The guide was prepared concerning the research question starting from general and moving to specific taking into consideration the local knowledge and cultural sensitivities. The guide developed to cover topics related to a) communities’ perception and experience in health-seeking behavior towards newborn illnesses, b) community-related barriers and facilitators (cultural and religious beliefs, awareness related to service availability, etc.), c) Health facility related barriers and facilitators; d) health extension-related barriers and facilitators of community-based management of possible serious bacterial infection.

All in-depth and key informant interview participants were communicated one before the data collection day. But, to FGD participants, they were recruited before 1 week, and communication was made before 3 days of data collection to select a suitable and comfortable setting for the discussion. Then, the interviews and FGDs were conducted at the participant’s natural setting. In-depth interviews with women who gave birth within 2 months before data collection and whose newborn was died were conducted at their home; interviews conducted with health workers, HEW, kebele chairman, and religious leader were conducted at their office, and FGDs were conducted within their community. The interviews were conducted only with the principal investigator while FGDs were conducted; the research assistant was used as note-taker and an audio-recorder. The principal investigator has used the guide during modulating the interviews and FGDs to cover all relevant topics. At the beginning of each FGD and IDI, the purpose of the study and topic of the discussions was mentioned to study participants, and then individual-based written informed consent was taken for their willingness to participate and also for recording their voice. On average, the FGDs lasted from 1:15 to 1:41 h and the interviews with community members lasted from 21:33 to 43:51 min and interviews with health workers lasted from 0:39:40 to 1:12 h.

### Data analysis

Inductive thematic analysis through which codes, categories, and themes are generated from the data was employed to analyze the data. The analysis was carried out simultaneously with data collection. After each data collection, debriefing of data was conducted with a research assistant to ensure data completeness and consistency with field notes. Simultaneously, data were analyzed to extract major themes, to plan for the next data collection, and discussions were also conducted with a research assistant to ensure data saturation. The data were begun to saturate after seven interviews and three FGDs were conducted. Then, the data were transcribed verbatim (in Afan Oromo and Amharic languages) from audio-recorded material. Ensuring the completeness and consistency of transcriptions, the data were translated to the English language by the principal investigator.

Then, important concepts that are related to the research question were extracted from the data after reading and re-reading the translations, and the codebook was developed. To develop the codebook, line by line coding was conducted separately by the principal investigator on ATLAS.Ti.7.1 software package, and one other peer who acts as a second research assistant on Microsoft word starting with richest data. After checking the inter-coder consistency, the codebook manual was developed to ensure code consistency, and credibility. Then, using the developed codebook, the whole data were coded by principal investigator coded the whole translations with simultaneous checking of intra-coder consistency. The potential categories and themes were developed by clustering sub-categories and categories, respectively, which answers the research question. Coding was repeated four times while refining the codebook, categories, and themes. Finally, findings were presented with two major themes, thirteen categories and quotations derived from the data concerning critical steps in the pathway: (a) community-related barriers and facilitators (b) health system-related barriers and facilitators.

### Trustworthiness (rigor)

To keep the trustworthiness of this study, different techniques were used. First, the guide was pre-tested with three women who gave birth 2 months before data collection and three health workers [two HEW and one under-five clinic focal] who reside at the neighboring district. Second, diversified study participants who have adequate experience in the area of interest/issue were recruited. Third, data were triangulated by collecting through interviews and focused group discussions from those diversified study participants. Forth, peer debriefing was done with a research assistant and research team. Fifth, at the end of each data collection period, a summary of major themes was raised for study participants, and discussion was conducted to clarify unclear concepts. Sixth, the transcriptions, translation, and findings were shared with key informants such as HEWs, focal persons of under-5 clinics, director of a health center and district health office, and MNCH expert to check the interpretations and to provide their comments, critiques, clarification, and confirmation. Seventh, through negative case analysis, contradicting ideas or deviant cases that emerged in the data was analyzed by enquiring deep information from potential study participants on the consecutive data collection periods. Eight, to ensure transferability, the whole research process, participant’s diverse perspectives and experiences, methodology, interpretation of results, and contributions of research assistants were thickly described. Professionals interested to apply the findings reported in this study may consider the transferability of the results after careful consideration of contextual information described earlier in the study setting section. Furthermore, the findings of the current study suit for the current Ethiopian primary health care structure and training system for health extension workers. Hence, analysis of contextual similarities is needed before taking up of the results of the current study to other contexts.

Ninth, to ensure dependability, the participant’s recruitment process, data collection methods, and the analysis process were clearly described. A detailed chronology of research activities and processes [data collection and analysis, emerging themes, categories or quotations] were audited by advisors, colleagues and other experts having good experience of qualitative research to confirm the procedures and verify whether they were used appropriately, and to make both the process and the study output consistent. Thus, with these activities, the process through which findings were derived was made explicit enough.

Tenth, confirmability of the study was ensured through different techniques. The first technique was the research team’s self-reflectivity and bracketing. The principal investigator is a public health officer in his background that has experience in working at a health center with different departments including under-5 clinics. Also, he had attended different pieces of training related to Community-Based Newborn Care, including management of newborn possible serious bacterial infection, had worked as CBNC project coordinator, and participated in different supportive supervisions, and performance review, and clinical mentoring meeting (PRCMM). Currently, he has a Master of Public Health in Health Promotion and Human Behavior. Besides this, he has also good experience in qualitative research. This preconception, knowledge, and skills benefited the principal investigator to set and focus on research questions. Other research teams have educational backgrounds in the health disciplines and have philosophical Degree [GTF] and Master of Public Health in Health Promotion and Human Behavior [YKL] and both have good experience of qualitative researches.

The study context and actual location of the research setting were different from where the principal investigator and research team are working. Therefore, there was no potential bias that could be introduced if they would be from the same location. However, since biases are not inevitable, as much as possible, subjectivity was managed by balancing together with the data, analytic processes, and findings in such a way that the reader can confirm the adequacy of the findings. Besides, the research teams speak the local language well. The research assistant has a good orientation to the local culture. This background used to minimize interpretation bias. Moreover, the interpretation of the findings was cross-checked by other peers by reading direct quotations from study participants. The second technique used to ensure conformability was through an audit trail. The findings of this study were audited and verified by colleagues and other experts having experience in qualitative research. The findings were also verified by key informants like HEWs, village leaders /kebele chairman, and health workers who participated in the study. Each study process was documented and audio records were available for cross-checking.

The third technique was a prolonged engagement. By spending enough time in the study setting and through creating rapport with study participants, the principal investigator observed and confirmed the findings of the interviews and FGDs. He had observed and understood like the closure of health posts [HP] on working hours, the short-time stay of HEWs in the health posts, HEWs traveling to and from the district town, punctuality of HEWs, presence or absence of arrangements such as pregnant women conferences, presence or absence of supervision and mentoring for HEWs, etc. Besides, he was also carefully reviewed the 0–2 month sick newborn registration book at six health posts and verified that many sick newborns were registered, assessed, classified and managed from 2013 to 2017, but there were few from 2017 till the data collection time.

## Result

### Participant’s socio-demographics

The demographic characteristics of participants are summarized in Table 1. The mean age was 37.6 years (range: 21–73 years). From all these participants fourteen of them were women who delivered within 2 months of data collection. The majority of them the participants were females, married, housewife, rural in residence, and age ranging from 31 to 40 years. All of them were Ethiopian Orthodox Christianity followers and Oromo in ethnicity.

**Table 1:** Demographic information of participants in Debre Libanos District, Oromia regional state, Ethiopia, 2019

Variable	Category	N (%)	Variable	Category	N (%)
Respondent type	Mothers who gave birth (0–2 month)	15 (28.8)	Education status	Illiterate	21 40.4)
	Health workers	7 (13.5)		Primary	20 (38.5)
	Religious leader	1 (1.9)		Secondary	4 (7.7)
	Pregnant women	5 (9.6)		Diploma	6 (11.5)
	Mother-in-law	6 (11.5)		Degree	1 (1.9)
	Care-givers of delivered women (0–2 month)	11 (21.2)	Occupation	Housewife	33 (63.5)
	Kebele chairman	1 (1.9)		Farmers	9 (17.3)
	Others^a^	6 (11.5)		Merchant	2 (3.8)
Age	20–30	17 (32.7)		HEW	1 (1.9)
	31–40	23 (44.2)		Health worker	4 (7.7)
	41–50	7 (13.5)		Kebele chairman	2 (3.8)
	51–60	3 (5.8)		Priest	1 (1.9)
	61–70	1 (1.9)	Residence	Urban	7 (13.5)
	> = 71	1 (1.9)		Rural	45 (86.5)
Sex	Male	16 (30.8)	Religion	Orthodox	52 (100)
	Female	36 (69.2)	Ethnicity	Oromo	52 (100)
Marital status	Single	4 (7.7)	N^o^_−_ of children under all participants	0	8 (15.4)
	Married	47 (90.4)		1–3	30 (57.7)
	Widowed	1 (1.9)		≥4	14 (26.9)

^a^Parents of a child treated at HP and HC, families of delivered women, father-in-law, and other reproductive age group peoples

The findings of the study are summarized based on two major themes and thirteen categories which are described below (Table 2). Except for the availability of trained human resources, monthly performance review meetings, and logistics mentioned as facilitators, most of the factors described can be both facilitators and barriers. If their absence is a barrier, their presence can be a facilitator for the implementation of the guideline. So, we did not want to make a demarcation between the two (between barriers and facilitators) while presenting them.

**Table 2:** Summary of barriers and facilitators for the successful implementation of community-based newborn possible serious bacterial infection management in Debre Libanos District, North Shoa zone, Oromia regional state, Ethiopia, 2019

Major themes	Categories
Community-related facilitators and barriers	Communities’ perception towards newborn illness: Perception of no treatment
	Communities’ perception towards newborn illness: Perception of non-severity and self-resolution
	Belief on the healing power of traditional medicines
	Awareness about the availability of the service at the health post
	Socio-cultural and religious beliefs.
Health system-related facilitators and barriers	Equipped human resource
	Shortage of Health extension workers
	Supervision, monitoring, and evaluation of activities
	The functionality of health developmental army
	Residence of health extension workers
	Health workers commitment
	Availability of logistics [medical supplies and job aids]
	Budget constraint

### Community-related facilitators and barriers

This theme contains a description of barriers related to the community members and caregivers that affect community-based service utilization for newborn PSBI management. It has five categories: communities’ perception of newborn illness (perception of no treatment, and non-severity and self-resolution), belief on the healing power of traditional medicines, awareness about the availability of sick newborn treatment at the health post, and socio-cultural and religious belief.

### Communities’ perception towards newborn illness: perception of no treatment

Participants mentioned that community members in this study setting locally diagnose newborn illnesses as a sun or hot burn [Mitch], body dislocation and or fracture [kichitat], demon [megagna], evil eye [buda], berd, tonsillitis [enlarged or dropped uvula] and common cold when they manifested with certain unspecified symptoms. For some of these illnesses diagnosed and named as such illnesses, they perceive that treatment is not needed at all or they perceive that they don’t be treated at all at health facilities. For example, for newborn illnesses locally named as body dislocation and or fracture [‘*Kichitat’], ‘megagna’, ‘berd’ and evil eye [‘buda’],* they perceive as there are no medications from health facilities unless they are treated locally by traditional medicines. Therefore, for such illnesses, they do not seek health care from health facilities [Table 3].

“*… for kichitat there is no medication [at health facilities] rather we take them to traditional healers [wogesha] and massaged. (22 years old, female, IDI participant, delivered mother).*

**Table 3:** Summary of local names of newborn illnesses, their perceived causes, symptoms, and mode of management in Debre Libanos District, North Shoa, Oromia regional state, Ethiopia, 2019

Newborn illness	Description	Symptoms	Treatment options
*Sun or hot burn [locally called mitch]*	From exposure to day time sunlight, [*qeter* time from 10 AM-5 PM], or immediate wearing of cloth stayed on sunlight and contact the care givers body immediately after staying around the fire or on sunlight.	Anyone or combination of symptoms: feeling hot, unable to breastfeed, vomiting, cough, irritability, body weakness, unconscious, skin rash, diarrhea, difficulty, or fast breathing.	First treatment option: Traditional medications prepared from the leaf of local herbs like *demakesse (*Ocimum lamiifolium)*, baharzaf (Eucalyptus globulus*)*, kebericho (*Echinops kebericho) and *tunjit (*Otostegia fruticosa). ‘*Demakesse’* is applied on the external body; make him/her to drink by punching and diluting with water or steaming with boiled water or by smoking on the fire. Similarly, the others are steamed. If not improved taken to the health facility.
*Body dislocation or fracture [kichitat]*	Newborn illness denoted to body dislocation or fracture from poor newborn handling. During this time they perceptive that lungs, heart, and intestine of the newborn dislocated or their neck or shoulder might be fractured.	Any one or combination of symptoms: irritability, vomiting, unable to breastfeed, groaning, change in diarrhea, fast breathing, feeling hot, and cough.	First treatment option: Traditional bone setter [*wogesha*] massages the body of the newborn using butter. If they do not improve, others like medications for *Mitch* will be provided to them or taken to a health facility.
*Berd*	Illness resulted from exposure to cold air/weather.	Cough plus with any of symptoms like fast breathing, crying, unable to breastfeed, irritability, groaning, chest in drawing, and diarrhea.	First treatment option: Covering with a cloth and frequent breastfeeding. There is nothing done for them until baptism [date of ‘*Kristina’*]. If not improved or gotten worse, taken to the health facility.
*Enlargement or dropping of uvula or tonsil [tonsillitis]*	Newborn illness resulted from the dropping of the brain [moves down]. Newborns might have a sore throat as a result of excessive crying.	Any one or combination of symptoms like unable to or difficulty of breastfeeding, vomiting, feeling hot, weakness, and frequent crying.	First treatment option: Treat traditionally by sucking the backside of the newborn neck or putting traditional medications on their head. With these, they perceive that the dropped brain returns to its normal size. Also might be taken to the health facility.
*Megagna*	Newborn illness which happens when the devil touches them.	Crying suddenly, paralyzing legs or hands, and other symptoms of evil eye sickness.	First treatment option: Treated traditionally by smoking *tunjit.* For protection, newborns would not be left alone, and sharp things are put beside them.
*Evil eye [locally called buda]*	Resulted from exposure to a person possessing an evil eye.	Anyone or combination of symptoms: unable to breastfeed, unable to open eyes, crying, irritable, loss of consciousness, body weakness, and difficulty of breathing.	First treatment option: Treated using traditional medications prepared from *xenadam (Ruta chalepensis), white onion* (*Allium sativum*)*, the root of grawa* (*Withania somnifera*) and *shiferaw (moringa olifera).* Provided in the form of putting around the nose to smell it, steamed by smoking on fire or dilute the medications and make them drink little by little.
*Common cold*	Newborn illness that occur from poor hygienic condition of the newborn or transferred from a caregiver.	Cough plus any of combination of symptoms like feeling hot, unable to breastfeed, fast breathing, wheezing, unable to open eye, grunting.	First treatment option: Treat it using home-based remedies prepared from *zingibil* [*ginger*] and *xenadam* added into boiled milk, and also by breastfeeding. If not improved taken to the health facility.

Study participants mentioned that care is sought from health facilities for such newborn illnesses if the newborn does not get better with traditional medicines or if they changed their diagnosis to other illnesses than these. Study participants also mentioned that for illnesses named locally as ‘megagna’, they do not provide any medication before baptism. This is because, for example, participants mentioned that community members primarily use holy water, even if others use other traditional medicines for treating demon, but since these newborns do not reach their age of ‘Kristina’ [baptism] and it is not allowed to use holy water to treat sick newborns before their date of baptism, they do not provide it for them until that day [Table 3].

“*… there is nothing done until they reach their 40 days [males newborn] or 80 days [female newborn]...” (42 years old, male, IDI participant, religious leader).*

### Communities’ perception towards newborn illness: perception of non-severity and self-resolution

Participants mentioned that community members perceive newborn illnesses as simple or non-severe that would resolve spontaneously by self within a few days. Therefore, they seek to care for their sick newborns when they fail to get better or if the condition worsens.

*“In our culture, there is a habit of simplifying things when newborns become sick. This is our habit. But, newborns less than two months would become sick on the first one or two weeks …*” *(32 years old, female, FGD participant, caregivers of delivered mother).*

### Belief on the healing power of traditional medicines

Community members in this study setting mainly use traditional medicines for treating their sick newborns. Participants mentioned that they use traditional medicines until sick newborns are taken to health facilities or newborns would be taken to health facilities when they do get better by traditional medicines [Table 3].

*“... If newborns cry, we suspect kichit and take him to wogesha [traditional bone setter]. Then, if the wogesha sees newborns and diagnosis it as a problem other than kichit, such as mitch, they would return home and provide medication for mitch … But, if a newborn does not get better with treatment by wogesha or medication of mitch, we would take the newborn to health facility …*” *(51 years old, female, FGD participant, mother-in-law).*

### Awareness about the availability of sick newborn treatment service at the health post

Participants involved in this study mentioned that they do not have awareness about the availability of sick newborn treatment at health post unlike that of immunization, 2–5 month treatment service or maternal services; or mentioned that they do not seek health care for their sick newborns due to lack of awareness about the availability of sick newborn treatment service at a health post. The reasons mentioned for this were limited attention given to the program, poor commitment among health workers, and unavailability of health extension workers at health posts on working hours.

“*… I do not know about the availability of newborn treatment there. I do not also think that such treatment is available for this kind of newborns.” (21 years old, female, IDI participant, delivered women).*

### Socio-cultural and religious beliefs

Socio-cultural and religious beliefs were mentioned as a barrier to the service utilization mentioned by study participants. Two socio-cultural and religious beliefs were mentioned. The first is that among the Orthodox Christian follower community members, newborns who have not reached their baptism date [‘*Kristina’]* are not taken out of home for any issue. Therefore, among these community members, it is not allowed to take newborns out of the home before their 40th day [for male newborns] or 80th day [for female newborns] for seeking care or other purposes. For example, a delivered mother before 2 months of data collection, in IDIs, reported that it is forbidden to take newborns out of home before date of Baptism [Christianization] for seeking treatment or other issues whether the illness or issue regardless of its severity.

“*… I couldn’t go because it is forbidden to take a newborn outside home before her Kristina* [baptism] day *… whether there is a problem or not …*” *(22 years old, female, IDI participant, delivered mother).*

The second socio-cultural and religious belief which affected the service utilization is ‘Hamechissa’ [Afan Oromo language]. Study participants mentioned that community members who believe in this culture do not take the newborns to the health facility for seeking care or any issue before they are taken to the ‘witch’ and he or she blesses the newborn. For example, health worker, in IDI, mentioned that there are some community members who follow this belief and not seek any treatment or other health service before they are taken to witch and blessed.

“*… At some kebeles, even to take for baptism, there is something called “hamechissa”. At those kebeles, newborns even do not taken out of home for getting treatment service, vaccine or not celebrate their Kristina* [baptism] *before going to “hamechissa” and the witch blesses them … “(30 years old, male, IDI participant, health worker).*

The reason for such a socio-cultural belief is that fear of illness from an evil spirit. If newborns are taken out of the home before their date of baptism and taken to PNC, getting treatment or if celebrated their date of baptism before they are taken to the ‘witch’ and he or she blesses them, the community members perceive that the newborn would face different illnesses from evil spirits. For example, husbands of delivered woman, in FGD, reported that if these conditions happen, the newborn would get illness from evil spirit or others.

*“ … The fear is that if the newborns are taken out of home, since she is small, it is said that the baby would face an evil eye … “ (34 years old, male, FGD participant, husbands of delivered woman).*

### Health system-related facilitators and barriers

This theme contains a description of barriers and facilitators related to the availability of trained health staffs, shortage of health extension workers, supervision, monitoring and evaluation of the activity, availability of logistics [medical supplies and job aids], the functionality of health developmental armies, budget constraint, HEWs and health workers commitment, and residence of HEWs that are related to the health system. These contents are well described below under eight categories.

### Equipped human resource

This study found that all rural HEWs who are currently on job had taken basic CBNC training during starting of project implementation. Study participants also mentioned that there is also one district health office expert who had taken CBNC orientation and two health workers trained on IMNCI from each health center. Unavailability of CBNC trained health staff that monitors this activity from health centers and district health offices was mentioned as a barrier that contributed to the discontinuation of CBNC program implementation. For example, one health worker, in IDI, reported that there is no available trained health worker at district health office who has enough knowledge, skill or experience to supervise, monitor or evaluate the program.

*“ … The reason for not conducting this is that at the woreda [district] level, no one knows about CBNC due to the unavailability of trained manpower.” (34 years old, male, IDI participant, health worker).*

### Shortage of health extension workers

In this study setting, two HPs have only one HEW per each health post and also there is one health post that has no HEW. This happened due to the transfer of HEWs to other health posts and due to resignation. Due to this case, participants mentioned that HPs are closed on working hours which affected community-based service utilization. For example, one community member, in FGD, reported that at some health posts who have only one; means that there is no enough HEW to provide the service for the community members at those kebeles.

*“...For this big kebele, we have only one health extension worker... How can one health extension worker reach, and create awareness among all the community members in this kebele? There is only one health extension worker. Within the kebele, if she goes to the other site, what about others who come here [HP]?” (34 years old, male, FGD participant, community member).*

### Supervision, monitoring, and evaluation of the program

Health care providers mentioned that the program was initiated and being implemented for 3 years in support of non-governmental organizations and implementing partners. At that time they mentioned that there was regular supportive supervision, monitoring and performance review, and clinical mentoring meeting [PRCMM]. Nevertheless, the program implementers were handover all he activities to the district health office for the last 3 years. Due to this problem, there was no supportive supervision, monitoring, or PRCMM conducted. Different reasons like a budget constraint, lack of CBNC trained health workers from health centers and district health office, lack of integrating health center staff during program implementation, lack of commitment among health facility directors, health care providers, and HEWs were mentioned by participants for the problem. For example, health worker, in IDIs, mentioned that sustainability of the treatment service was affected due to unavailability of trained man power from district health office, budget problem or commitment of HEWs.

*“The reason for not sustaining treatment services for newborns is that at the District level, there is no one who knows about CBNC in detail due to the unavailability of trained manpower … To support these activities specifically, there is a budget problem … On HEWs there is a problem like that of commitment and burnout.” (34 years old, male, IDI participant, health worker).*

The other issue, study participants mentioned that there is periodic performance review meetings conducted at the health center and district health office levels with health extension workers. Even though this is present, they mentioned that there is no usage of data for decision making or regular [weekly-based] supportive supervision given to health extension workers unless there is immunization or periodic activities due to weak health center and health post linkage. For example, health workers mentioned that there were no program specific utilization of data for decision making, or regular supportive supervision conducted to enhance the service utilization due to weak health center and health post linkage.

*“ … Anyways the major problem within our home [health center] is, there is nothing done at a time when zero report or no activities were conducted.” (30 years old, male, IDI participant, health worker).*

*“ … PHCU [primary health care unit-health center] and health post have very week linkage. There is a gap that they did not go weekly to support, identify, and solve gaps of health workers [HEWs] unless there were other opportunities like campaign and periodic activities.” (34 years old, male, IDI participant, health worker).*

### The functionality of health developmental army

Participants mentioned that, currently, due to the non-functionality of health developmental army [HDA], all of health activity performances, including community-based management of PSBI among newborns, were low. Major reasons for its non-functionality mentioned by participants were weakness during their organization, no follow up or monitoring from HEWs, health workers, kebele command post, or district level concerned bodies like women league. For example, a health worker, in IDI, reported that HEWs activity was affected by non-functionality of health developmental armies.

“*… what makes their [HEWs] activity to be hindered is just not making organizations below them to be functional like health developmental armies.” (30 years old, male, IDI participant, health worker).*

### Availability of logistics [medical supplies and job aids]

Health workers mentioned that there were no medical supply or job aids related problems faced since the time of CBNC implementation. They mentioned that medical equipment is supplied or re-filled from the pharmaceutical fund and supply agency (PFSA) and zonal health department. A health worker, in IDI, reported that there was no any logistic problem faced to provide the service for 0–2 month newborns.

“*… when you see as an office [health center] or organization [health office], we do not have any supply or medication problem for both under two months and all under five.” (30 years old, male, IDI participant, health worker).*

### Budget constraint

Health center and district health office managers mentioned that budget constraints have deterred them in order from supervising or conducting PRCMM specifically for this program. For example, a health worker, in IDI, reported that there was budget constraint to provide follow-up or conduct review meetings for health extension workers.

*“ … there is a budget problem to go and visit all health posts and to pay Per diem for them if they conduct PRCMM for at least two days … It is impossible to cover all things by government budget … “ (34 years old, male, IDI participant, health worker).*

### Residency of health extension workers

In this study setting, throughout the interviews and group discussions, the most commonly mentioned issue by participants was that except two HEWs, all other rural HEWs live and work traveling from District towns due to the absence of residence home constructed for them within the kebele. Due to this case, participants mentioned that health posts are not opened on working hours or no service is given on weekends and holidays. Through this, community members mentioned as they faced challenges to utilizing the service from health posts. For example, fathers of delivered woman, in FGD, reported that health extension workers assigned at health did not live around the health post to facilitate service provision.

*“ … They [HEWs] are only available here for only two days. When they are not available here, we expense transport costs to go to town [health center]. Since they are assigned as a government employer, why do they live here and provide treatment service?” (32 years old, male, FGD participant, fathers of delivered women).*

### Health extension workers and health workers commitment

Study participants were mentioned that health workers from health centers and district health officials are not committed to supporting health extension workers through regular supportive supervision. On the other hand, the study participants were commonly mentioned that most of the time the health posts are closed throughout working hours over a week. They also mentioned that there are HEWs who open health posts for a maximum of 3 days per week. On the days when the health extension workers are available at the health posts, they might not reach health posts on time or do not stay full working hours of a day. This is because they travel from district town where they live. For example, participants mentioned that HEWs might not reach even till 4 or 5 AM [local time] or returns early at around 8 PM [local time]. For example, health workers mentioned that HEWs were available for a maximum of 3 days, and they did not stay at health posts on full working hours of over a course of a week.

*“ … It [HP] might be open once per week … The health posts only open for the EPI program but not for other activities. We are not expecting newborns would get such treatment with this condition.”(32 years old, male, IDI participant, Health worker).*

Due to this issue, the study participants were mentioned that HEWs are not conducting their routine activities like providing PNC service, conducting pregnant women conference, promoting the availability of services, and providing treatment at health posts. They were also mentioned that HEWs do not provide services intentionally unless it is during the EPI program.

“*… During EPI time, they go there and provide postnatal service for those who delivered at home and as well for mothers who returned after delivery at the health facility. As I have told you it is given at home during this time rather going purposively for it.” (30 years old, male, IDI participant, health worker).*

## Discussion

This study found the availability of trained manpower, logistics [medical supply and job aids], and monthly performance review meetings as facilitators for community-based service utilization for newborn PSBI management. Also, barriers such as communities perception that newborn illnesses have no medical treatment, perception of non-severity and self-resolution, belief in the healing power of traditional medicines, socio-cultural and religious beliefs, lack of awareness on the availability of sick newborn treatment service at the health post, lack of program supervision, monitoring and evaluation, shortage of HEWs, shortage of trained health workers, the residence of HEWs, poor health workers and HEWs’ commitment and non-functionality of health developmental army were explored.

This study found that community members locally diagnose newborn illness as the sun or hot burn (‘mitch’), body dislocation or fracture (‘kichitat’), the evil eye (‘buda)’, ‘megagna’ (locally perceived as a newborn illness caused by demon-evil spirit), ‘berd’ (locally perceived as a newborn illness caused by exposure of coldness), common cold and tonsillitis (enlarged or dropped uvula) from perceived but misconceived causes of illnesses. This local illness diagnosis makes the community members perceive that newborn illnesses have no medical treatment from health facilities, and rely on the traditional medicines rather than seeking care from health facilities; developing misconception on treatment options. These findings are consistent with the findings of studies conducted in central and southern Ethiopia, Nigeria, and Bangladesh in that community members use herbal medicine to treat newborn illnesses [23–28]. This study also found that community members perceive newborn illnesses as non-severe which resolve spontaneously within a few days. This finding is consistent with findings from different studies conducted at different settings in that community members in those settings delay sought health care from health facilities due to expectation of self-resolution [28, 29] and considering the symptoms as minor that resolve within next few days [28, 30]. This calls a need to conduct a behavioral change communication to change the behavior of the community members towards newborn illnesses and their treatment.

Lack of awareness on the availability of sick newborn treatment at a health post among the community members is one explored barrier in this study. This was because awareness creation or promotion of the availability of sick newborn service at health posts for the community members was not done due to lack of commitment, unavailability of HEWs at health posts on working hours, and non- functionality of health developmental armies in the study setting that facilitate service utilization. But, community empowerment and demand creation is one key objective to create awareness and promote service for effective use of newborn and child survival interventions in Ethiopian newborn and child survival programs [17]. Therefore, this underscores the importance of conducting awareness creation activities for the community members to utilize the service for their sick newborns.

This study also explored socio-cultural and religious beliefs as a barrier for the community-based service utilization for newborn PSBI management. Community members who follow the Ethiopian Orthodox Christian religion do not take their newborns out of home for seeking care before the date of baptism even if the newborn is severely sick. On the other hand, community members who believe in ‘*hamachisa’ (a local culture through which the community members take their newborns to the witch and he or she blesses them).* In both beliefs, if newborns are taken out of home for seeking treatment, PNC, or other issues before their date of baptism or taken to the witch for blessing, community members believe that newborns would face sickness from evil spirits or others. This finding is similar to the finding of a study conducted in Ethiopia which showed tradition recommends newborns to stay at home for 40 days because they are vulnerable to malevolent spirits [27, 31]. This also underscores the need to conduct a behavioral change communication to change the behavior of the community members towards newborn illnesses and their treatment.

This study also found that there were no program-related supportive supervisions, monitoring, or evaluations conducted for the last 2–3 years after the implementing partner was phased-out. In contrast, the program is expected to be monitored through integrated supportive supervision twice a month, program-focused supervision once per month, and PRCMM twice a year [18]. This happened from lack of giving attention to the program from health facilities and lack of commitment among health care providers. This implies that there are weak health center and health post linkage [32]. This study also found that there are two health workers trained on IMNCI from each health center, and all rural HEWs who attended CBNC training which meet the expectation [18].

This study found that two HPs have only one HEW, and one HP with no HEW which happened due to transfer and resigning. In contrast, according to the Health Extension Program, two health extension workers should be assigned per each health post [31]. This makes difficult to conduct activities in static or outreach program. This calls a need to assign two health extension workers at these health posts. The finding this study also showed that except two HEWs, all other rural HEWs live at and travel from the district town due to lack of constructed home at assigned kebeles. In contrast, residence in the village is one of the HEW recruitment criteria [33], and CBNC also acknowledges the importance of available HEWs close to the community to provide gentamycin injection for newborns with PSBI for 7 days [18]. But, since the HEWs live at and traveling from the district town, health posts were not open all days of working hours or service is not given for sick newborns on working hours, weekends, holy days, or night time. The health posts are open for a maximum of 3 days per week and less than 5 hours per day. Therefore, this study finding is lower from the study findings which showed that approximately 15 % HPs were open less than 5 days of the week, and also over half of HEWs serve the community weekends or holidays [18]. Similarly, this finding is lower when compared with the finding of an observational time-motion study conducted which showed that HEWs were on duty for an average of 15.5% and they stayed on duty for about 6 hours per day [34].

A poor commitment of health extension workers to conduct post-natal care and pregnant women conferences also identified as a barrier for the community-based service utilization for newborn PSBI management. This is because both these activities are used as an entry point to promote the service, get sick newborns, and provide treatment service. Specifically, the postnatal care (PNC) is essential for teaching caregivers how to recognize sick newborns, screening or identifying sick newborns and provide care on the riskiest periods, first day, and the week of life. Thus, HEWs are responsible to provide a home to home PNC service on the first, third, and seventh day to identify and treat illnesses in newborns with amoxicillin and gentamycin [9, 18, 27]. On the other hand, conducting pregnant women conference is used to promote the service and facilitates in developing health care seeking newborn illnesses [28]. Nevertheless, the result from this study showed that there were no pregnant women conducted for the last 3 years, and HEWs did not provide PNC service on these critical days which might result in addressing sick newborns to screen for danger signs and sign and symptom of PSBI. This underscores the importance of providing attention to provide post-natal care to address sick newborns.

Lastly, this study found that there were non-functional health developmental armies from poor supervision or monitoring given by health extension workers. But, the implementation of the Health extension program is facilitated when HEWs are conducting activities in support of health developmental armies. This is because they play a substantial role in increasing the healthcare-seeking behavior of the community regarding MNCH services, promoting the availability of services delivered at the community and health facility level including ICCM, CBNC, skill birth, etc. [17]. There were also evidences that health developmental armies made remarkable achievements concerning pregnant women identification, providing ANC and PNC counseling service, sick newborn identification, referring sick children to health posts, and promoting the service [18]. Nevertheless, the result of this study showed that there the availability of non-functional health developmental armies. Due to these issues, study participants mentioned that community-based management of newborn PSBI was low.

### Strength and limitation

The strength of this study is the involvement of participants from different socio-demographic backgrounds, the use of mixed data collection techniques and exploring barriers and facilitators at community, health facilities, and health care provider level have been explored. The potential limitation of this study is that there might be recall bias since it explored the participant’s experience.

## Conclusions

The findings of this study provides insight into health care providers and community member’s view on the facilitators of and barriers to the community-based service utilization for newborn PSBI management, that calls a need to develop strategies that fit the local context to take action to address the explored barriers. Therefore, health care providers and concerned bodies should have to develop the best fit strategies, and conduct a behavioral change communication to change the perception of community members towards newborn illnesses, and develop their care-seeking behavior. Furthermore, health extension workers should have to promote the availability of the service at the health posts, and provide the service staying at the health post.

## Data Availability

All study data were reported in the table.

## Abbreviations

ANC: Antenatal care
CBNC: Community Based Newborn Care
FGD: Focused group discussion
HAD: Health developmental army
HC: Health Center
HEW: Health Extension Worker
HP: Health Post
IDI: In-depth interview
IMNCI: Integrated Management of Newborn and Childhood illness
MDG: Millennium development goal
MNCH: Maternal, Neonatal and child health
PFSA: Pharmaceutical fund and supply agency
PHCU: Primary Health Care Unit
PNC: Post-natal care
PRCMM: Performance review and clinical mentoring meeting
PSBI: Possible serious bacterial infection
WHO: World Health Organization
